# Medication Adherence Status and Its Association With Quality of Life Among Individuals With Neurological Conditions in Saudi Arabia

**DOI:** 10.7759/cureus.40508

**Published:** 2023-06-16

**Authors:** Omar Babateen, Sarah S Aldharman, Ghazi Mogharbel, Ahmad S Badawi, Sultan A Aljohani, Manar M Alsharif, Marwah S AL-Jallal, Jamil A Samkari

**Affiliations:** 1 Department of Physiology, Umm Al-Qura University, Makkah, SAU; 2 College of Medicine, King Saud Bin Abdulaziz University for Health Sciences, Riyadh, SAU; 3 College of Medicine, Taibah University, Medina, SAU; 4 College of Medicine, Umm Al-Qura University, Makkah, SAU; 5 College of Medicine, King Khalid University, Abha, SAU; 6 Department of Family and Community Medicine, King Abdul Aziz University, Jeddah, SAU

**Keywords:** adherence, medication, saudi arabia, neurological conditions, quality of life

## Abstract

Introduction: Chronic disorders commonly require long-term therapies. Medication non-adherence can cause major morbidity and mortality in chronic illness individuals, as well as increase the financial burden on the healthcare system. It is considered that patients who adhere to their treatment may improve their quality of life (QoL). There is a scarcity of updated comprehensive data on medication adherence among Saudi patients with neurological disorders. Therefore, this study aimed to assess the medication adherence status among individuals with neurological conditions and its association with QoL.

Method: A cross-sectional questionnaire-based study was conducted. The study included subjects individuals who have neurological conditions aged at least 18 from different regions of Saudi Arabia. The questionnaire measured medication adherence by using the 10-item version of the Medication Adherence Report Scale (MARS-10, ©Professor Rob Horne). The QoL was measured by employing validated Euro Quality of Life 5-dimension scale (EQ-5D).

Results: A total of 370 participants were included. Respondents aged 18 to 35 years represented 62.4% of the sample. More than half of the participants were females (65.7%). The most frequently reported chronic conditions were migraine (29.2%), epilepsy (20.8%), and multiple sclerosis (20.5%). The reliability of the EQ-5D questionnaire was acceptable (Cronbach's alpha = 0.764). In general, more than half of the participants indicated that had problems due to pain/discomfort (60.3%) and anxiety/depression (62.2%). The most common pattern of non-adherence was taking the medication only when a patient needed it followed by avoiding taking the medication as possible. Non-adherence to medications was less prevalent among participants with epilepsy (68.8%) and multiple sclerosis (65.8%). On the other hand, medication adherence was higher among respondents with migraine compared to participants without the condition (86.1% vs 73.7%, p = 0.009). A significantly lower proportion of participants who had some or extreme problems with self-care were non-adherent to medications compared to those who had no problems (68.1% vs 80.3%, respectively, p = 0.016). Results of the regression analysis showed that participants with epilepsy and multiple sclerosis were less likely to be non-adherence to medications. Furthermore, respondents with moderate and severe problems in self-care were less likely to be non-adherent.

Conclusion: It was found that more than half of the participants had problems regarding their QoL due to pain/discomfort and anxiety/depression. The most prevalent pattern of non-adherence was taking the medication only when needed. Participants with epilepsy and multiple sclerosis were less likely to be non-adherent to medications. Furthermore, respondents with moderate and severe problems in self-care were less likely to be non-adherent. We recommend serial studies on the issue should be conducted to gather more evidence regarding this topic.

## Introduction

Adherence has been defined as "the patient's active, willing, and cooperative participation in a mutually acceptable course of behavior that leads to a therapeutic result" [1]. This means that the patient has a choice and that the treatment goals and medical plan are set by both the patient and the provider [1]. Medication adherence usually means whether a patient takes their medicine as prescribed (for example, twice a day) and whether or not they keep taking the medicine they were prescribed. Therefore, the behavior of taking medications as prescribed has been broken down into two main ideas: adherence and persistence [2]. Even though the ideas are similar, adherence refers to how much a person uses a drug during therapy, while persistence refers to how long a person stays on drug therapy [2].

Even though some studies have found a connection between not taking medications as prescribed and poor outcomes, there is some concern that this connection may be due, at least in part, to a "healthy adherer" effect [3]. The healthy adherer effect describes a phenomenon in which patients who adhere to medical therapies are more likely to engage in health-seeking behaviors. Post-hoc analyses of randomized, controlled clinical trials have shown that even adhering to a placebo is linked to better results than for patients who don't adhere to active treatment [4].

Medication adherence is the process by which patients take their medications as prescribed by their caregiver [2]. It is divided into three stages: initiating treatment, maintaining the treatment plan, and discontinuing treatment [5]. Up to 50% of patients don't follow the instruction regarding their medications [5]. About 25-66% of people who should take antiepileptic drugs (AEDs) don't [6]. Also, more people with epilepsy in Malaysia (64.1%) don't take their AEDs as prescribed than in the West [7].

Some studies explored the associations between medication adherence and quality of life (QOL) [8]. The literature also highlighted that people with epilepsy have a lower QOL and more psychosocial problems than the general population [9]. A study using a meta-analysis found the prevalence of anxiety (20.2%) and depression (22.9%) in epilepsy were similar [9]. A study looked at medication adherence among hypertensive stroke patients after discharge in China and found that medication adherence among hypertensive stroke patients was poor [10]. Non-adherence leads to medication waste, disease progression, decreased capacity to function, reduced QOL, more hospital visits, and hospital hospitalizations [11].

Medication adherence plays a major role in the QOL of patients with neurological diseases such as epilepsy. Medication non-adherence has been found to be high among individuals with neurological diseases [12]. There was a significant difference in QOL between adherent and non-adherent individuals [12]. Medication compliance affects patients' QOL, health costs, and, indirectly, their caregivers [13]. Another study demonstrates a significant correlation between physical health and medication compliance in multiple sclerosis patients [14]. In the United States, a study targeting epilepsy patients and their adherence showed that poor adherence correlates with poor symptomatic control and a lower QOL [15]. A systematic review done on Parkinson’s disease concluded that QOL is lower in non-adherent patients with advanced disease [16]. Locally in Saudi Arabia, a cross-sectional study evaluating medication adherence and its effect on the QOL amongst epileptic patients in the eastern side of the country concluded that a better state of QOL is present in patients with moderate to high adherence to medications [17]. Another study that investigated treatment satisfaction and adherence among multiple sclerosis patients showed that high satisfaction was associated with better adherence [18].

Medication non-adherence can cause major morbidity and mortality in chronic disease patients, as well as increase the financial burden on the healthcare system. The prevalence of neurological illnesses is rising in Saudi Arabia; yet there is a scarcity of comprehensive data on medication adherence among Saudi patients with these conditions. Therefore, this study aimed to assess the medication adherence status among individuals with neurological conditions and its association with QoL in Saudi Arabia.

## Materials and methods

Data collection method

The study was a descriptive cross-sectional questionnaire-based study. Data was collected through an online self-administered questionnaire. It was distributed on different online platforms to collect data from different regions of Saudi Arabia.

Study populations

Our target population of this study was individuals who have neurological conditions (multiple sclerosis, amyotrophic lateral sclerosis, Parkinson’s disease, muscular dystrophy, Alzheimer’s disease, stroke, benign brain tumors, epilepsy, myasthenia gravis, motor neuron disorders, and other neurological conditions) and aged at least 18 from different regions of Saudi Arabia (Central, Southern, Eastern, Western, North).

The inclusion criteria were for both genders aged at least 18 years old. Also, individuals should have neurological conditions. Participants with incomplete questionnaires or do not agree to participate were excluded.

Sampling technique and sample size

The representative sample size required is 385 determined by using the Raosoft sample size calculator (Raosoft, Inc., Seattle, USA) available online with a margin error determined as 5%, confidence level determined as 95%, and the population of Saudi Arabia determined as 34,000,00. The data will be collected by convenience sampling technique.

Data collection tools

The questionnaire consisted of three main sections. The first section consisted of the participant's personal information. The second section measured medication adherence by using the 10-item version of the Medication Adherence Report Scale (MARS-10, ©Professor Rob Horne). It is a self‐report adherence scale that evaluates both intentional (“I avoid using it if I can”) and nonintentional nonadherence (“I forget to use it”) [19,20]. It is intended to obtain patient reports of nonadherence, a strategy that has previously been validated in research [21,22]. The questionnaire is reliable in assessing nonadherence in a variety of conditions [19,23]. Respondents were asked to rate how frequently they engaged in each behavior associated with adherence on a five-point scale (5 = never, 4 = rarely, 3 = sometimes, 2 = often, and 1 = always). A total score was calculated by adding the scores for each item, with higher scores representing a higher degree of reported adherence. Self-reported adherence is reported as the average of the 10 questions (range of 0 to 5, higher numbers indicate greater adherence). A MARS score of 4.5 or higher was considered to indicate high self-reported adherence. The third section consisted of QoL which was measured by employing validated Euro Quality of Life 5-dimension scale (EQ-5D) [24]. The EQ-5D questionnaire examines five dimensions of health-related QoL (mobility, self-care, usual activities, pain/discomfort, and anxiety/depression) utilizing three levels (no problems, some/moderate problems, and extreme problems). Participants select one statement that best reflects their health across all five dimensions. A distinct health condition is established by combining level one from each of the five domains.

Data presentation and statistical analysis

Ten items of the MARS were used to assess medication non-adherence among the participants. The coding of two statements was reversed such that the overall score expresses the non-adherence state; these statements were "I take the medication regularly every day" and "I take the medication exactly as prescribed". The reversed statements were coded as 1 = Never to 5 = Always. The overall score of non-adherence was computed by calculating the average of the responses. Therefore, the non-adherence score ranged between 1 and 5. The MARS score of < 4.5 indicated non-adherence.

Regarding the QoL, participants' responses to the EQ-5D were collected on a three-point scale, including no problem, some problem, or extreme problem. A distinct health condition was considered by combining the moderate and severe responses into a single category named "some problem".

The survey was provided electronically using Google Forms. The participant was asked if he/she has been diagnosed with any of the following neurological conditions (multiple sclerosis, amyotrophic lateral sclerosis, Parkinson’s disease, muscular dystrophy, Alzheimer’s disease, stroke, benign brain tumors, epilepsy, myasthenia gravis, motor neuron disorders, or other neurological conditions). If there is any of these were chosen, the participant continue the questionnaire. However, if he/she answered (No), the survey automatically ends for him/her.

After finishing filling out the surveys, they were checked for completeness. The collected data were first entered into a Microsoft Excel file and later transferred to Statistical Product and Service Solutions (SPSS) (IBM SPSS Statistics for Windows, Armonk, NY) for further analysis. All information was confidential and was only used for scientific research, and participation in this research was voluntary and optional with informed consent on the first page. The study was conducted in agreement with the principles of the Declaration of Helsinki and all participants were informed of the nature and the objectives of the study at the beginning of the survey. The ethical approval for the study was obtained from the biomedical research ethics committee at Umm Al-Qura University (approval no. HAPO-02-K-012-2022-11-1252).

Statistical analysis

Frequencies and percentages were used to present the categorical data. The internal consistency of the EQ-5D and MARS scales was assessed using Cronbach's alpha values, and an acceptable value was set at an alpha value of > 0.7. Factors associated with the non-adherence to medications were assessed using Pearson's Chi-squared test or Fisher's exact test whenever applicable. The significantly associated variables in the inferential analysis were used as independent variables in a multivariable binary logistic regression model to assess the independent predictors of non-adherence. Results were presented as odds ratio (OR) and the respective 95% confidence intervals (95% CIs). The p-value of < 0.05 indicated statistical significance.

## Results

Sociodemographic characteristics of the respondents

Initially, we collected 649 responses on the online platform. However, we excluded two responses from those who disagreed to participate. A total of 276 responses from those who had not been diagnosed with neurological conditions, and one participant with missing responses to the MARS questions. Therefore, the current study included data is 370 participants. Respondents aged from 18 to 35 years represented 62.4% of the sample. More than half of the participants were females (65.7%) and had obtained a Bachelor's degree (54.3%), and less than half of them were married (45.9%). About one-third of them had a monthly income of 5,000 to 10,000 SAR (Table 1).

**Table 1:** Sociodemographic characteristics

Parameter	Category	N (%)
Age (years)	18 to 25	113 (30.5%)
	26 to 35	118 (31.9%)
	36 to 45	66 (17.8%)
	46 to 55	31 (8.4%)
	> 55	42 (11.4%)
Gender	Male	127 (34.3%)
	Female	243 (65.7%)
Marital status	Single	166 (44.9%)
	Married	170 (45.9%)
	Widow	10 (2.7%)
	Divorced	24 (6.5%)
Education	Public education	143 (38.6%)
	Bachelor	201 (54.3%)
	Postgraduate studies	26 (7.0%)
Monthly family income (SAR)	< 5,000	101 (27.3%)
	5,000 to 10,000	117 (31.6%)
	10,000 to 20,000	101 (27.3%)
	> 20,000	51 (13.8%)
Number of medications used	1	163 (44.1%)
	2	99 (26.8%)
	3	57 (15.4%)
	4	23 (6.2%)
	> 4	28 (7.6%)

Characteristics of the neurological conditions

**Figure 1:**
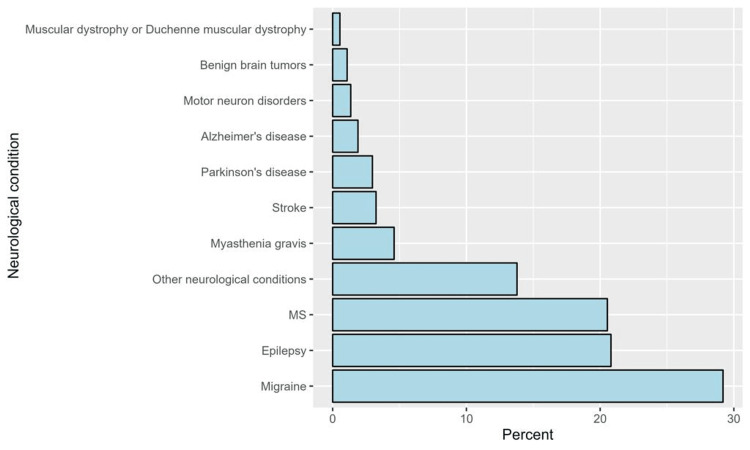
The percentages of neurological conditions among participants under study MS: multiple sclerosis

The most frequently reported chronic conditions were migraine (29.2%), epilepsy (20.8%), and multiple sclerosis (20.5%), whereas the least common conditions were muscular dystrophy (0.5%) and benign brain tumors (1.1%) (see Figure 1).

Responses to the EQ-5D questionnaire

The reliability of the questionnaire was acceptable (Cronbach's alpha = 0.764). In general, more than half of the participants indicated that had problems due to pain/discomfort (60.3%) and anxiety/depression (62.2%). Additionally, less than half of them had problems in usual activities (45.7%), mobility (39.7%), and self-care (24.6%) (Table 2).

**Table 2:** Participants' responses to the quality-of-life items

Parameter	Category	N (%)
Mobility	I have no problems in walking about	223 (60.3%)
	I have some problems in walking about	137 (37.0%)
	I am confined to bed	10 (2.7%)
Self-care	I have no problems with self-care	279 (75.4%)
	I have some trouble washing or dressing myself	77 (20.8%)
	I am unable to wash or dress myself	14 (3.8%)
Usual activities	I have no problems with performing my usual activities	201 (54.3%)
	I have some problems with performing my usual activities	146 (39.5%)
	I am unable to perform usual activities	23 (6.2%)
Pain/Discomfort	I have no pain or discomfort	147 (39.7%)
	I have moderate pain or discomfort	195 (52.7%)
	I have extreme pain or discomfort	28 (7.6%)
Anxiety/Depression	I am not anxious or depressed	140 (37.8%)
	I am moderately anxious or depressed	173 (46.8%)
	I am extremely anxious or depressed	57 (15.4%)

Responses to the MARS questionnaire

Regarding the non-adherence domain, the internal consistency was adequate as indicated by a Cronbach's alpha of 0.795. The most common pattern of non-adherence was taking the medication only when a patient needed it (46.8% responded as always) followed by avoiding taking the medication as possible (10.3% responded as always). Conversely, changing the medication dose and taking the medication less than instructed were the least common patterns of non-adherence (2.4% and 3.2% responded as always, respectively) (Table 3).

**Table 3:** Participants' responses to the MARS questionnaire MARS: Medication Adherence Report Scale

Item	Always	Often	Sometimes	Rarely	Never
I take the medication only when I need it	173 (46.8%)	63 (17.0%)	57 (15.4%)	21 (5.7%)	56 (15.1%)
I take the medication regularly every day	193 (52.2%)	69 (18.6%)	54 (14.6%)	21 (5.7%)	33 (8.9%)
I avoid taking the medication if I can	38 (10.3%)	43 (11.6%)	75 (20.3%)	55 (14.9%)	159 (43.0%)
I forget to take the medication	17 (4.6%)	34 (9.2%)	106 (28.6%)	112 (30.3%)	101 (27.3%)
I alter the dose of the medication	9 (2.4%)	25 (6.8%)	63 (17.0%)	70 (18.9%)	203 (54.9%)
I stop taking the medication for a while	26 (7.0%)	38 (10.3%)	69 (18.6%)	57 (15.4%)	180 (48.6%)
I take the medication exactly as prescribed	242 (65.4%)	52 (14.1%)	50 (13.5%)	6 (1.6%)	20 (5.4%)
I miss out on a dose of the medication	21 (5.7%)	32 (8.6%)	107 (28.9%)	112 (30.3%)	98 (26.5%)
I take the medication more than instructed	17 (4.6%)	13 (3.5%)	32 (8.6%)	50 (13.5%)	258 (69.7%)
I take the medication less than instructed	12 (3.2%)	21 (5.7%)	34 (9.2%)	57 (15.4%)	246 (66.5%)

Factors associated with non-adherence to medications

Non-adherence to medications was less prevalent among participants with epilepsy (68.8% vs 79.5%, p = 0.046) and multiple sclerosis (65.8% vs 80.3%, p = 0.007) compared to their peers without neurological conditions. On the other hand, medication adherence was significantly more prevalent among respondents with migraine compared to participants without the condition (86.1% vs 73.7%, p = 0.009) (Table 4). Of note, a significantly lower proportion of participants who had some or extreme problems with self-care were non-adherent to medications compared to those who had no problems (68.1% vs 80.3%, respectively, p = 0.016). Other QoL items did not differ significantly between adherent and non-adherent participants (Table 5).

**Table 4:** Differences between adherence and non-adherent participants in terms of their sociodemographic and clinical history-related characteristics

Parameter	Category	Adherent, N = 84	Non-adherent, N = 286	p-value
Age (years)	18 to 25	22 (19.5%)	91 (80.5%)	0.222
	26 to 35	35 (29.7%)	83 (70.3%)	
	36 to 45	14 (21.2%)	52 (78.8%)	
	46 to 55	7 (22.6%)	24 (77.4%)	
	> 55	6 (14.3%)	36 (85.7%)	
Gender	Male	30 (23.6%)	97 (76.4%)	0.760
	Female	54 (22.2%)	189 (77.8%)	
Marital status	Single	38 (22.9%)	128 (77.1%)	0.357
	Married	42 (24.7%)	128 (75.3%)	
	Widow	2 (20.0%)	8 (80.0%)	
	Divorced	2 (8.3%)	22 (91.7%)	
Education	Public education	25 (17.5%)	118 (82.5%)	0.135
	Bachelor	51 (25.4%)	150 (74.6%)	
	Postgraduate studies	8 (30.8%)	18 (69.2%)	
Monthly family income (SAR)	< 5,000	17 (16.8%)	84 (83.2%)	0.299
	5,000 to 10,000	26 (22.2%)	91 (77.8%)	
	10,000 to 20,000	28 (27.7%)	73 (72.3%)	
	> 20,000	13 (25.5%)	38 (74.5%)	
Number of medications used	1	36 (22.1%)	127 (77.9%)	0.397
	2	23 (23.2%)	76 (76.8%)	
	3	12 (21.1%)	45 (78.9%)	
	4	3 (13.0%)	20 (87.0%)	
	> 4	10 (35.7%)	18 (64.3%)	
Epilepsy	No	60 (20.5%)	233 (79.5%)	0.046
	Yes	24 (31.2%)	53 (68.8%)	
Multiple Sclerosis	No	58 (19.7%)	236 (80.3%)	0.007
	Yes	26 (34.2%)	50 (65.8%)	
Myasthenia gravis	No	80 (22.7%)	273 (77.3%)	>0.999
	Yes	4 (23.5%)	13 (76.5%)	
Stroke	No	82 (22.9%)	276 (77.1%)	>0.999
	Yes	2 (16.7%)	10 (83.3%)	
Alzheimer's disease	No	83 (22.9%)	280 (77.1%)	>0.999
	Yes	1 (14.3%)	6 (85.7%)	
Parkinson's disease	No	84 (23.4%)	275 (76.6%)	0.076
	Yes	0 (0.0%)	11 (100.0%)	
Benign brain tumors	No	83 (22.7%)	283 (77.3%)	>0.999
	Yes	1 (25.0%)	3 (75.0%)	
Muscular dystrophy	No	84 (22.8%)	284 (77.2%)	>0.999
	Yes	0 (0.0%)	2 (100.0%)	
Motor neuron disorders	No	81 (22.2%)	284 (77.8%)	0.079
	Yes	3 (60.0%)	2 (40.0%)	
Migraine	No	69 (26.3%)	193 (73.7%)	0.009
	Yes	15 (13.9%)	93 (86.1%)	
Other neurological conditions	No	76 (23.8%)	243 (76.2%)	0.198
	Yes	8 (15.7%)	43 (84.3%)	

**Table 5:** Differences between adherence and non-adherent participants in terms of their responses to the quality-of-life domain

Parameter	Category	Adherent, N = 84	Non-adherent, N = 286	p-value
Mobility	No problem	46 (20.6%)	177 (79.4%)	0.241
	Some Problem	38 (25.9%)	109 (74.1%)	
Self-care	No problem	55 (19.7%)	224 (80.3%)	0.016
	Some Problem	29 (31.9%)	62 (68.1%)	
Usual activities	No problem	40 (19.9%)	161 (80.1%)	0.161
	Some Problem	44 (26.0%)	125 (74.0%)	
Pain/Discomfort	No problem	32 (21.8%)	115 (78.2%)	0.728
	Some Problem	52 (23.3%)	171 (76.7%)	
Anxiety/Depression	No problem	26 (18.6%)	114 (81.4%)	0.139
	Some Problem	58 (25.2%)	172 (74.8%)	

Predictors of non-adherence to medications

Results of the regression analysis showed that participants with epilepsy (OR = 0.41, 95% CI, 0.20 to 0.82, p = 0.013) and multiple sclerosis (OR = 0.32, 95% CI, 0.15 to 0.66, p = 0.002) were less likely to be non-adherence to medications. Furthermore, respondents with moderate and severe problems in self-care were less likely to be non-adherent (OR = 0.46, 95% CI, 0.26 to 0.81, p = 0.007) (see Table 6).

**Table 6:** Predictors of non-adherence to medications OR: odds ratio; CI: confidence intervals

Parameter	Category	OR	95% CI	p-value
Epilepsy	No	-	-	
	Yes	0.41	0.20, 0.82	0.013
Multiple Sclerosis	No	-	-	
	Yes	0.32	0.15, 0.66	0.002
Migraine	No	-	-	
	Yes	1.04	0.49, 2.26	0.913
Self-care	No problem	-	-	
	Some Problem	0.46	0.26, 0.81	0.007

## Discussion

Medication adherence in chronic diseases is recognized as an important public health problem because non-adherence to medicines can result in increased healthcare costs and poor health outcomes. This study aimed to assess medication adherence and its association with QoL among patients with neurological disorders. A total of 370 participants were included in the study. Among those, the most frequently reported chronic conditions were migraine (29.2%), followed by epilepsy (20.8%), and multiple sclerosis (20.5%). Besides being prevalent neurological disorders, most of the published studies regarding medication adherence targeted individuals with those exact disorders, specifically epilepsy, and multiple sclerosis.

Analysis of the responses to the QoL questionnaire revealed that more than half of the participants had problems due to pain/discomfort (60.3%) and anxiety/depression (62.2%). Similarly, a study in Rwanda reported that anxiety was highly experienced by patients with epilepsy who were not adherent to their medications [25]. This could be due to fear of experiencing seizures, especially if the attack is to occur in inconvenient places or situations (i.e., in a public place).

On the other hand, studies reported that patients who adhered to their treatment experienced an improvement in QoL and vice versa, as for a study conducted among epileptic patients [26], and another study among diabetic patients with type 2 diabetes [27]. This implies that good adherence to medications can provide good control of symptoms, resulting in an improved QoL.

Non-adherence to medications was less prevalent among participants with epilepsy and multiple sclerosis in this study. Potential barriers to non-adherence, as identified from the studies, included patient-related factors such as disease-related knowledge and health literacy; drug-related factors such as experienced adverse effects; and other factors including the patient-provider relationship and various logistical and financial barriers to obtaining medications [28]. The complexity of medication regimens was also reported to be associated with reduced treatment adherence [29]. This is attributed as well to health literacy and the complexity of neurological disorders, hence requiring multiple medication regimes and protocols, which could be hectic in terms of compliance and financial terms as well.

Considering predictors of medication non-adherence, regression analysis revealed that participants with epilepsy and multiple sclerosis were less likely to be non-adherent to medications (OR = 0.41, 95% CI, 0.20 to 0.82, p = 0.013 for epilepsy and OR = 0.32, 95% CI, 0.15 to 0.66, p = 0.002 for multiple sclerosis). This is in contrast with a previous study that reported 66.7% of epileptic participants were not adherent to their medications [12]. This could be due to the differences in adherence assessment methods, study design, study populations, and sample sizes across studies. In addition, people residing in different countries have their cultural norms and may prefer traditional medicines over modern regimes [30].

Limited numbers of Saudi studies were published in this scope; hence this study is considered a valuable base for evidence. Another strength of this study is that it included participants from variable demographical backgrounds and socio-economic statuses, which would aid the authorities in dealing with the issue from all aspects. The study was not without limitations. The fact that it was done within a small sample size may have determined a highly selected group of respondents. It may therefore be difficult to generalize the findings to the total community. Bigger numbers of respondents would have improved the statistical significance of the results.

There are some limitations in this study. The fact that it was done via online platforms may have determined a highly selected group of respondents. It may therefore be difficult to generalize the findings to the total community of patients with neurological diseases. Bigger numbers of respondents would have improved the statistical significance of the results. In addition, the type of medications that patients were taking, and the clinical characteristics of diseases were not recorded so it could not be related to medication non-adherence.

## Conclusions

It was found that medication non-adherence was common among individuals with neurological conditions. Also, more than half of the participants experienced anxiety or depression which has a negative impact on the QoL. The most common pattern of non-adherence was taking the medication only when a patient needed it. Participants with epilepsy and multiple sclerosis were less likely to be non-adherent to medications. We recommend that serial studies on the issue should be conducted to gather more evidence regarding this topic. Different media platforms are recommended to be recruited, as well as broadcasts and webinars to display educational materials to patients with neurological diseases.
